# Open label study of a novel fiber product for bloating, gas, and bowel regularity

**DOI:** 10.3389/fmed.2025.1572261

**Published:** 2025-03-27

**Authors:** Alexander S. Ganz, Brooke Greeley, James Riggen, Aundria Riggen

**Affiliations:** ^1^Department of Biology, Southern Methodist University, Dallas, NC, United States; ^2^Department of Biology, Indiana University, Bloomington, IN, United States; ^3^LX Medical, Edina, MN, United States

**Keywords:** gas, bloating, fiber, constipation, flatulence

## Abstract

**Introduction:**

This open-label study evaluated the effects of a novel fiber supplement product, Relievance, on bowel movement regularity and symptoms of bloating and gas in 20 consecutive patients. The product is a commercially available combination of 8 natural fibers and the recommended dosage is approximately 6 grams per serving.

**Methods:**

Participants, presenting with chronic bothersome gastrointestinal symptoms of bloating, gas or bowel irregularity were recruited from an outpatient primary care practice for the study. Participants consumed 6 g of the fiber blend (one heaping teaspoon), one to three times per day, over 6 weeks, and kept diaries on symptoms and number of bowel movements per week. Symptoms were assessed via a 6-point analog scale at baseline and at 6 weeks. Primary endpoints included changes in bowel movement frequency and symptom severity related to gas and bloating.

**Results:**

At baseline, participants averaged 1.7 bowel movements per week, which significantly increased to 3.2 per week by the study’s conclusion. Symptom severity scores for bloating and gas also improved significantly, decreasing from 3.0 at baseline to 1.5 after 6 weeks. These results demonstrated statistically significant improvements in both bowel regularity and symptom relief. No significant adverse events were reported during the study, highlighting the product’s safety profile.

**Conclusion:**

This small, non-randomized prospective trial suggests that Relievance, a proprietary blend of eight different fibers, may effectively enhance bowel movement frequency and alleviate symptoms of bloating and gas. Further randomized controlled trials are warranted to confirm these findings and explore broader applications.

## Introduction

Dietary fiber is a diverse group of plant-derived carbohydrates that resist digestion in the human small intestine and undergo partial or complete fermentation in the colon. Fiber is broadly classified into two categories: soluble and insoluble, based on its solubility in water. Soluble fiber, found in foods such as oats, beans, and certain fruits, dissolves in water to form a gel-like substance and can modulate glycemic control and lipid metabolism (1). Insoluble fiber, prevalent in whole grains and vegetables, adds bulk to stool and facilitates bowel regularity (2). Beyond these classifications, fermentable and non-fermentable fibers play a significant role in gut health, impacting microbial composition and metabolic byproducts such as short-chain fatty acids (SCFAs), which are vital for colonic health and systemic metabolic regulation (3).

The health benefits of dietary fiber extend beyond gastrointestinal function, influencing systemic outcomes such as cardiovascular health, glucose metabolism, and weight management (4). In the gut, fiber serves as a substrate for microbial fermentation, promoting a balanced microbiome and enhancing colonic motility. Soluble fibers, such as inulin and psyllium, have been shown to improve stool consistency and alleviate symptoms of constipation, while insoluble fibers primarily contribute to mechanical stimulation of peristalsis (5). Fiber consumption has also been associated with a reduced risk of colorectal cancer and inflammatory bowel diseases (6). However, excessive fiber intake or an imbalance in fiber types can lead to adverse effects such as bloating, gas, and abdominal discomfort, highlighting the need for an optimized combination of fiber types to maximize benefits while minimizing side effects (7).

Emerging research suggests that combining various fiber types can yield synergistic health benefits by targeting different aspects of gut physiology. A combination of soluble and insoluble fibers, along with fermentable prebiotic fibers, can optimize bowel habits, support microbiota diversity, and reduce gastrointestinal symptoms (8). Studies have indicated that specific combinations of fibers can alleviate symptoms such as bloating and gas by promoting a balanced fermentation process and enhancing gut motility. However, the optimal combination of fiber types and their relative proportions remain an area of ongoing investigation, necessitating further research to determine the most effective formulations for improving gut and systemic health outcomes (9).

The main objective of the present study was to assess the impact of a novel supplement containing a combination of eight different fiber types on gut symptoms, with a particular focus on bloating, gas, and bowel habit regulation. Utilizing a prospective, nonrandomized patient series, the study employed six-point analog questionnaires to evaluate patient-reported outcomes before and after supplement use. By systematically analyzing symptom changes, this investigation sought to provide valuable insights into the efficacy and tolerability of multi-fiber supplementation in improving gastrointestinal comfort and function.

## Methods

**Figure 1:**
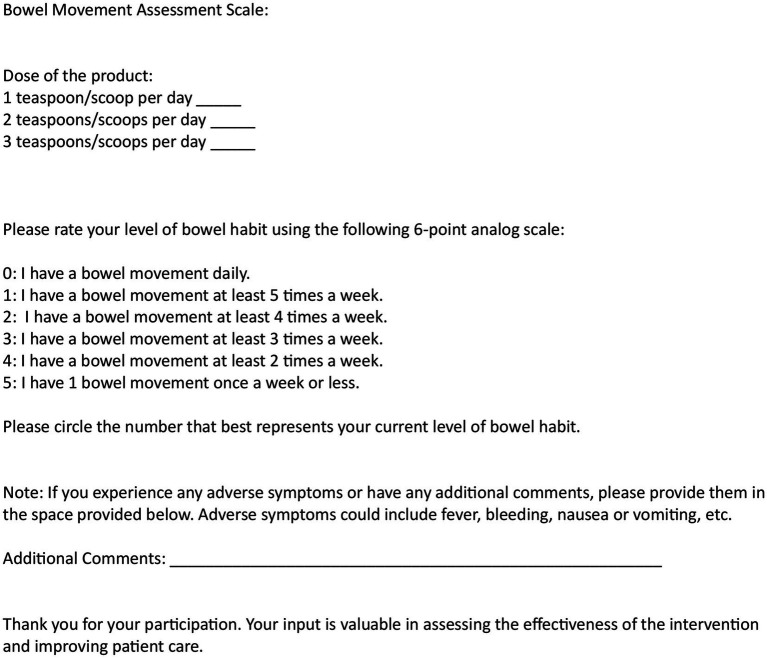
Bowel movement assessment scale. Study participants assessed number of bowel movements at baseline and at 6 weeks of product (Relievance) use via a 6-point analog scale. Dose of the product was also noted.

**Figure 2:**
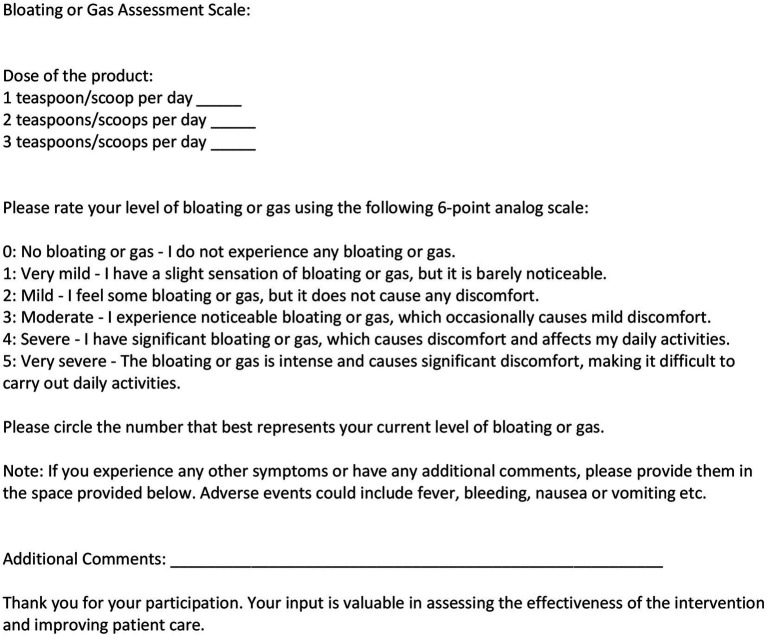
Bloating or gas assessment scale. Study participants assessed bothersome bloating and/or gas symptoms at baseline and at 6 weeks of product (Relievance) use via a 6-point analog scale. Dose of the product was also noted.

A prospective, nonrandomized, open label patient series with rolling admission was conducted to evaluate the effects of a novel fiber supplement, “Relievance,” on gastrointestinal symptoms, specifically bloating, gas, and bowel regularity. The primary objective of the study was to assess the impact of Relievance on bloating and gas symptoms, as well as bowel movement frequency. The primary endpoints included changes in self-reported bloating and gas severity, and the number of bowel movements per week while taking the supplement compared to baseline. Secondary endpoints included the assessment of any adverse events reported by participants throughout the study period (Figures 1, 2).

The study product, Relievance, is a proprietary, commercially available over-the-counter fiber powder supplement composed of a blend of eight different fibers. The fibers in Relievance include a combination of soluble, partially soluble, and insoluble fibers, with varying fermentability and viscosities. The supplement contains flaxseed, cellulose, guar gum, pectin, inulin, beta-glucan, resistant starch, and ispaghula. This formulation is designed to provide a comprehensive approach to gut health by combining fibers with different physical and chemical properties, promoting bowel regularity, supporting microbial diversity, and potentially reducing gastrointestinal symptoms such as bloating and gas.

A total of 20 outpatients were recruited from a single outpatient primary care medical practice (LX Medical, Edina, MN) and enrolled for a duration of 6 weeks. Any person with symptoms appropriate for fiber supplementation was eligible to participate. Potential participants were screened to determine their eligibility based on patient perceived bothersome gas or bloating, or bothersome bowel irregularity. Modified Rome criteria for functional constipation or constipation-predominant irritable bowel syndrome (IBS-C) were used to assess potential study participants but were not mandatory for entry into the study.

The study was IRB-approved by Western Copernicus Group (WCG-IRB, Princeton, NJ) and written informed consent was obtained from all participants before enrollment. Participants were instructed to take the supplement daily for 6 weeks, following the manufacturers recommended dosage.

Participants were allowed to continue all their typical care and lifestyle and were also allowed to continue all medications, prebiotics or fibers they were currently using. Any use of cannabis or cannabidiol (CBD oil) were also continued as taken.

Specific inclusion criteria included adults aged 18–90 who met one of the following:

- Subjects experienced two or more of the following criteria for at least 3 months with symptom onset at least 6 months prior:
  - Straining during at least 25% of defecations.
  - Lumpy or hard stools in at least 25% of defecations.
  - Sensation of incomplete evacuation for at least 25% of defecations.
  - Sensation of anorectal obstruction/blockage for at least 25% of defecations.
  - Manual maneuvers to facilitate at least 25% of defecations (e.g., digital evacuation, support of the pelvic floor)
  - Loose stools rarely present without the use of laxatives

Or,

- Subjects experienced 2 or more of the following criteria of recurrent abdominal pain, or bothersome gas or bloating, for at least 3 months, with symptom onset at least 6 months prior:
  - Pain, bloating or gas related to defecation.
  - Associated with a change in frequency of stool.
  - Associated with a change in form (appearance) of stools.

Or,

- Subjects experienced 2 or more of the following criteria for at least 3 months, with symptom onset at least 6 months prior:
  - Recurrent abdominal pain, gas or bloating, on average, at least 1 day per week in the last 3 months, associated with two or more of the following criteria:
  - Pain, bloating or gas related to defecation.
  - Pain, bloating or gas associated with a change in frequency of stool.
  - Pain, bloating or gas associated with a change in form (appearance) of stool.
  - Patients enrolled had symptoms that were chronic and interfered with daily activities, caused worry or affected the patient’s quality of life.

Exclusion criteria included:

- Loose stools present for more than 25% of defecations
- Unable to provide informed consent
- Hematochezia
- Prior colon surgery
- Thyroid disease
- Pelvic floor disorder
- Known allergies or intolerance to fiber products
- Infectious diarrhea or food poisoning
- Inflammatory bowel disease, including microscopic colitis
- Fecal incontinence or rectal prolapse

### Intervention

Participants were instructed to use the fiber product (Relievance) as currently labeled, with a dosage of 6 grams per day (one heaping teaspoon), initially taken once daily, increasing up to 3 heaping teaspoons a day (maximum dose 18 grams/day) on an as needed basis. The increased dose could be taken all at once or portioned throughout the day or evening per individual preference. The fiber product was mixed and stirred with any liquid of the participant’s choice.

### Assessments

Participants were prospectively assessed for symptoms at baseline and at 6 weeks of product use. The primary endpoints, number of bowel movements, and symptom severity of bloating and gas were assessed by number of bowel movements in the last 7-day period at baseline and at 6 weeks, by patient recall/diary. The co-primary endpoint, reduction in bloating or gas was assessed using analog scales, by 72-h recall at baseline and at 6 weeks. The secondary endpoint, adverse events, were recorded per patient report. Dosage amounts of daily product intake were also recorded (Tables 1, 2). Participants were advised to maintain their usual dietary and lifestyle habits to minimize confounding variables. Compliance with the supplement regimen was monitored through self-reported adherence logs. Patients were asked to document their daily symptom scores, noting any changes in bloating, gas, and bowel movement frequency, as well as any adverse effects experienced during the study period. To measure symptom severity and frequency, participants completed six-point analog scale questionnaires at baseline and after completion of the 6-week study period. The scales ranged from 0 (no symptoms) to 5 (severe symptoms), providing a standardized measure of subjective symptom burden (Figures 1, 2).

**Table 1:** Gas and bloating assessment.

Patient #	Age	Ethnicity	BMI	Inclusion criteria	Exclusion criteria	Dose 1–3 tsps/day	Initial symptoms	Follow-up symptoms	Adverse events
001	70	Caucasian	26.2	Yes	No	One	5	3	None
002	79	Caucasian	21.2	Yes	No	One	4	2	None
003	53	Caucasian	28	Yes	No	One	4	0	None
004	49	Caucasian	19.5	Yes	No	Two	3	0	None
005	55	Caucasian	21.7	Yes	No	One	2	1	None
006	54	Caucasian	22	Yes	No	One	1	1	None
007	53	Mixed race	19.9	Yes	No	One	4	3	None
008	21	Black	28	Yes	No	One	4	3	None
009	80	Caucasian	28.2	Yes	No	Two	2	0	None
010	65	Caucasian	29.8	Yes	No	One	3	2	None
011	70	Black	Unknown	Yes	No	One	2	1	None
012	76	Caucasian	33.6	Yes	No	One	3	4	None
013	21	Caucasian	25.0	Yes	No	Two	3	3	None
014	44	Caucasian	Unknown	Yes	No	Two	4	2	None
015	46	Caucasian	25.6	Yes	No	One	5	2	None
016	48	Caucasian	34.3	Yes	No	One	2	1	Hives
017	38	Caucasian	22.5	Yes	No	One	1	0	None
018	78	Caucasian	20.2	Yes	No	One	3	0	None
019	71	Caucasian	20.6	Yes	No	One	3	2	None
020	35	Caucasian	27	Yes	No	One	2	1	None

**Table 2:** Bowel movement assessment.

Patient #	Age	Ethnicity	BMI	Inclusion criteria	Exclusion criteria	Dose 1–3 tsps/day	BMs per week baseline	BMs per week post	Adverse events
001	70	Caucasian	26.2	Yes	No	One	0	2	None
002	79	Caucasian	21.2	Yes	No	One	2	4	None
003	53	Caucasian	28	Yes	No	One	0	4	None
004	49	Caucasian	19.5	Yes	No	Two	2	5	None
005	55	Caucasian	21.7	Yes	No	One	3	4	None
006	54	Caucasian	22	Yes	No	One	5	5	None
007	53	Mixed race	19.9	Yes	No	One	2	3	None
008	21	Black	28	Yes	No	One	3	4	None
009	80	Caucasian	28.2	Yes	No	Two	1	2	None
010	65	Caucasian	29.8	Yes	No	One	2	3	None
011	70	Black	Unknown	Yes	No	One	3	4	None
012	76	Caucasian	33.6	Yes	No	One	1	2	None
013	21	Caucasian	25.0	Yes	No	Two	3	3	None
014	44	Caucasian	Unknown	Yes	No	Two	2	4	None
015	46	Caucasian	25.6	Yes	No	One	1	4	None
016	48	Caucasian	34.3	Yes	No	One	2	3	Hives
017	38	Caucasian	22.5	Yes	No	One	0	1	None
018	78	Caucasian	20.2	Yes	No	One	0	3	None
019	71	Caucasian	20.6	Yes	No	One	2	3	None
020	35	Caucasian	27	Yes	No	One	0	1	None

### Safety monitoring

Adverse events (AEs) were monitored throughout the study period. Participants were instructed to report any adverse events or concerns to the study team and were given a number to call in the event of any adverse events. Depending on the adverse event and the study team determination, subjects were instructed to discontinue the supplement. In addition, depending on the adverse event, and the study team determination, subjects could be referred to their primary care physician to assess any new or worsening symptoms after using the supplement.

### Data collection

Participant demographics, data entry and adverse events were recorded. Any diary entries for bowel movements and amount of daily product taken were collected. Analog scale measurements for bloating or gas reduction were collected.

### Data analysis

Descriptive statistics were used to summarize participant characteristics. The primary endpoints, number of bowel movements per week, and symptoms of bloating and gas were analyzed using the Student’s paired t-test, with an alpha value of 0.05, and pre-test probability of 30% improvement in number of bowel movements per week or 30% improvement in bloating or gas symptom scores. The study employed a pre-post design wherein the patient data was naturally paired, as each patient served as their own control. The paired T-test is specifically designed for such studies, analyzing within-subject differences rather than between-group comparisons. Adverse events were tabulated as a percentage of events per total number of participants in the study. The sample size of 20 patients was determined based on the feasibility of recruitment within the study duration and power analysis. Given an expected 30% improvement in symptoms or bowel movements per week, and a *p*-value of 0.5%, the study was adequately powered to detect said effects. All statistical analysis was performed using appropriate software (e.g., SPSS, SAS). As noted, a *p*-value <0.05 was considered statistically significant.

## Results

A total of 20 patients were consecutively enrolled in the study. Of these, 17 (85%) were Caucasian, 2 (10%) were Black, and 1 (5%) was of mixed race. The study population was 65% female and 35% male, with an age range of 21 to 80 years. Baseline body mass index (BMI) ranged from 19.9 to 34.3 kg/m2.

Sixteen participants took either one heaping teaspoon (80%), and four took 2 heaping teaspoons (20%) of the fiber supplement daily. The supplement was well tolerated, with 19 of 20 participants (95%) reporting no adverse events. One participant (5%) developed hives within 1 week of starting the product, however, the relationship to the supplement was unclear. The hives resolved within 24 h without medication, and this participant completed the study protocol without further issues. (Tables 1, 2).

**Figure 3:**
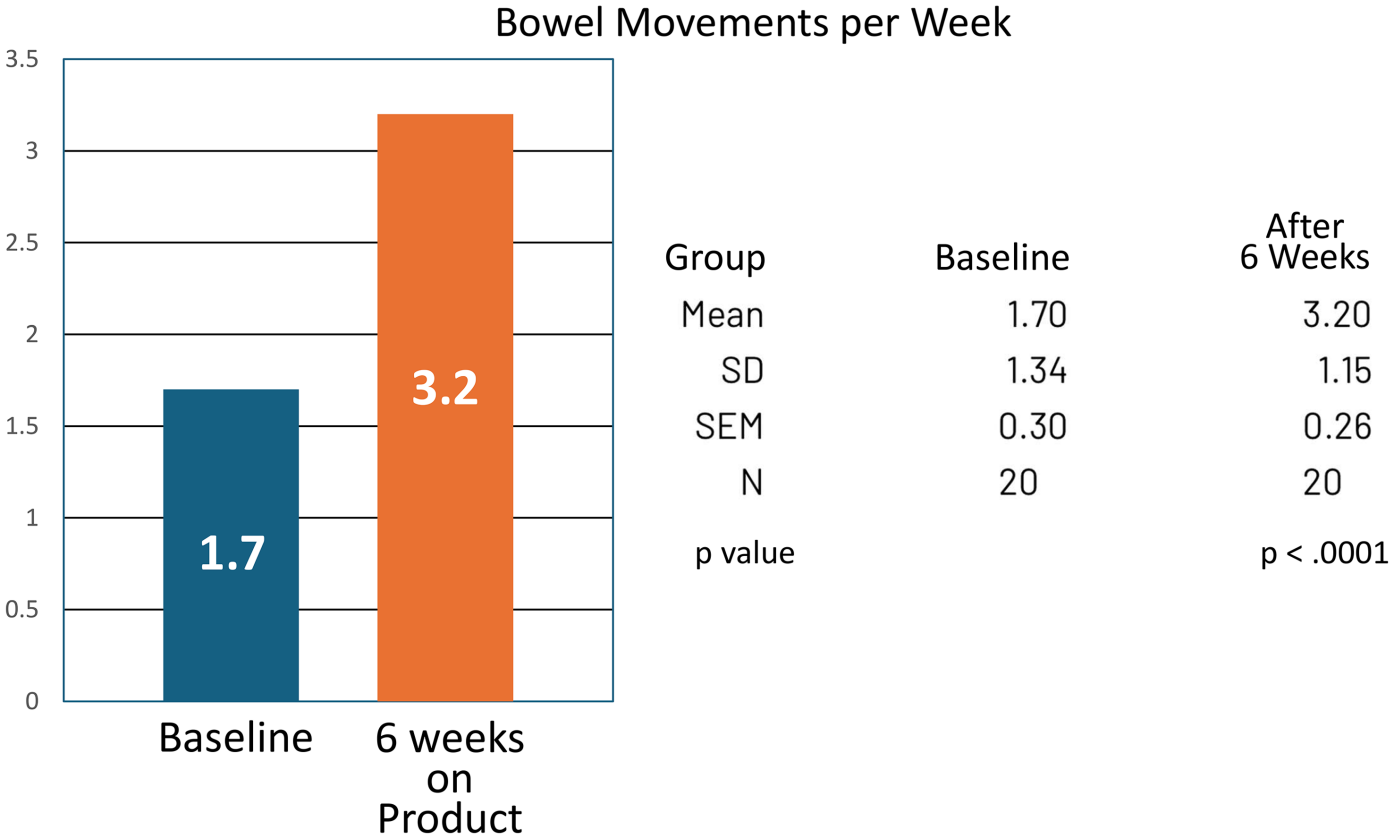
Bowel movements per week at baseline and after 6 weeks of product (Relievance) use. The increase in bowel movements from 1.7 to 3.2 per week was highly statistically significant (*p* < 0.0001).

**Figure 4:**
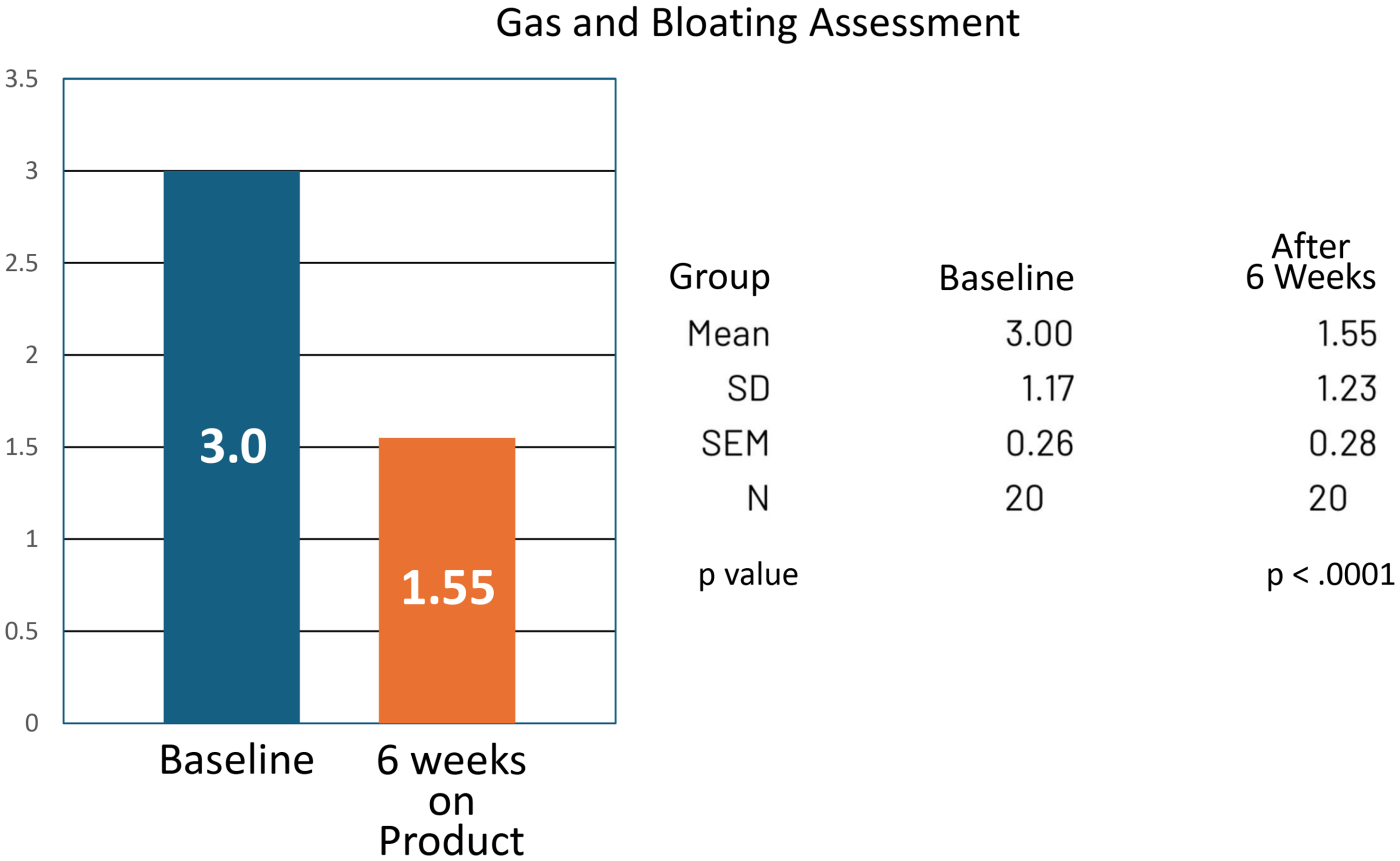
Gas and bloating symptom assessment at baseline and at 6 weeks of product (Relievance) use. The decrease in symptoms at 6 weeks was highly statistically significant (*p* < 0.0001).

For the group, bowel movement frequency increased from a mean of 1.70 per week at baseline to 3.20 per week at the end of the study (CI −1.99 to −1.01; *p* < 0.0001). Reported symptoms of bloating and gas decreased, with mean analog scores improving from 3.00 at baseline to 1.55 at study completion (CI 0.89–2.01; *p* < 0.0001) (Tables 1, 2; Figures 3, 4).

## Conclusion

Dietary fiber plays a crucial role in human health, supporting digestive function, metabolic regulation, and overall well-being. The inclusion of fiber in daily diets has been shown to promote gut health, improve bowel regularity, and reduce symptoms such as bloating and gas. Combining different types of fibers with varying solubility, fermentability, and viscosity can offer synergistic benefits, enhancing gastrointestinal comfort and promoting a balanced gut microbiota. Research suggests that fiber combinations can support a more diverse gut microbiome compared to single fiber sources, as different fibers serve as substrates for distinct microbial populations (10). A diverse gut microbiome has been linked to improved digestive function, enhanced immune response, and reduced risk of chronic diseases such as obesity, diabetes, and inflammatory conditions (11).

The key findings in this small, nonrandomized prospective series, are that use of Relievance, a proprietary blend of eight different fibers, was associated with a significant reduction in bloating and gas symptoms, with participants reporting an improvement of at least 30% based on six-point analog scale assessments. Additionally, bowel regularity improved by an average of more than one spontaneous bowel movement per week, indicating the potential efficacy of the supplement in enhancing gut motility and function. Importantly, Relievance was well tolerated, with no significant adverse events reported, and participants found the product easy to take as part of their daily routine. These findings suggest that the diverse fiber composition in Relievance may not only help alleviate gastrointestinal symptoms but may also contribute to microbiome diversity, which could have significant systemic health benefits.

Despite these promising results, the nonrandomized design and relatively small sample size of this patient series present limitations that should be considered. The absence of a control group limits the ability to attribute observed improvements solely to the supplement, and self-reported outcomes may have introduced recall bias. Placebo effects cannot be ruled out, and existing supplement use may have impacted results as well. Future randomized, placebo-controlled, double blind trials with larger sample sizes, stool consistency scores and longer follow-up periods are needed to further validate these findings and establish Relievance’s long-term efficacy and safety. Future studies could also potentially include standardizing baseline dietary intake and a microbiome analysis to see the effect of this supplement on the gut microbiome might be insightful as well.

Overall, this study provides encouraging preliminary evidence that Relievance may be a beneficial, well-tolerated, and convenient intervention for individuals experiencing bloating, gas, and irregular bowel habits. The potential of combination fiber supplements to promote a more diverse gut microbiome may offer additional systemic health benefits beyond gastrointestinal symptom relief. While the results are promising, further research is warranted to confirm these findings and explore the broader implications of multi-fiber supplementation in improving digestive and overall health. Future studies are planned to build on this initial work and further elucidate the role of fiber diversity in gastrointestinal and systemic health management.

## Data Availability

The original contributions presented in the study are included in the article/supplementary material, further inquiries can be directed to the corresponding author.
